# Choice of Treatment for Patients With Non–small-cell Lung Cancer >5 cm Between Surgery Alone and Surgery Plus Adjuvant Radiotherapy

**DOI:** 10.3389/fsurg.2021.649802

**Published:** 2021-03-09

**Authors:** Boyan Wang, Yongjie Zhou, Min Jia, Zhiping Yan, Jiayan Chen, Xueguan Lu, Ruiyan Wu, Junmiao Wen

**Affiliations:** ^1^Department of Radiation Oncology, Fudan University Shanghai Cancer Center, Shanghai, China; ^2^Department of Oncology, Shanghai Medical College, Fudan University, Shanghai, China; ^3^Institute of Thoracic Oncology, Fudan University, Shanghai, China; ^4^Department of Interventional Radiology, Zhongshan Hospital, Fudan University, Shanghai, China

**Keywords:** NSCLC, surgery, postoperative radiotherapy, node-negative, T-stage

## Abstract

**Background:** According to the lung cancer staging project, T2b (>5–7 cm) and T3 (>7 cm) non-small cell lung cancers (NSCLC) should be reclassified into T3 and T4 groups. The objective of this study was to evaluate the effect of surgery alone or surgery plus adjuvant radiation (SART) on survival of node-negative patients with NSCLC >5 cm.

**Methods:** We identified 4557 N0 patients with NSCLC >5 cm in the Surveillance, Epidemiology, and End Results database from 2004 to 2014. Overall survival (OS) and cancer–specific survival (CSS) were compared among patients who underwent surgery alone and SART. The proportional hazards model was applied to evaluate multiple prognostic factors.

**Results:** 1,042 and 525 patients who underwent surgery alone and SART, respectively were enrolled after propensity-score matching. OS and CSS favored surgery alone rather than SART. Multivariate analysis showed that the number of lymph nodes examined more than six was associated with better OS and CSS for NSCLC >5 cm, especially in patients treated with surgery alone. Lobectomy should be recommended as the primary option for NSCLC >5 to 7 cm, whereas its superiority was not significant over sublobectomy for NSCLC >7 cm.

**Conclusion:** Surgery alone should be recommended as the first choice for patients with NSCLC >5 cm. The number of examined lymph nodes should be more than six in patients with NSCLC >5 cm, especially for those who undergo surgery alone. For patients with NSCLC >7 cm who could not tolerate lobectomy, sublobectomy might be an alternative surgical procedure.

## Introduction

Lung cancer is the leading cause of cancer death and the second most prevalent cancer in both men and women in the United States (1), with ~222,500 estimated new cases in 2017 (1). Non-small cell lung cancer (NSCLC) constitute the most common type of lung cancer (2). Surgery with or without chemotherapy has been adopted as the main treatment offered for curative intent among patients presenting with early-stage disease, and multimodality consultation has become particularly important for curative-intent treatment of locally advanced NSCLC (3) (stage II-III disease).

The optimal treatment strategy for large pulmonary tumors remains uncertain. The International Association for the Study of Lung Cancer (IASLC) proposed a significant change on T descriptor in the eighth edition of the TNM classification for lung cancer in 2015 (4), in which tumors >5 cm to less than or equal to 7 cm were reclassified as T3, and those greater than 7cm as T4 (4). The proposal has been adopted in the 8th edition of the American Joint Committee on Cancer (AJCC) and the Union for International Cancer Control (UICC) staging system. Notably, stage IIB disease includes T3 tumors > 5 cm with no lymph node extension (T3N0), while stage IIIA includes T4 tumors >7 cm without lymph node involvement (T4N0). However, there has not yet been specific study focusing on the optimal treatment modality for patients with NSCLC > 5 to 7 cm and > 7 cm based on the latest TNM staging system.

Surgery plus adjuvant radiotherapy has been considered an important treatment for locally advanced lung cancer (5). However, postoperative radiotherapy (PORT) was routinely not recommended for patients with pathologic stage N0 or N1 disease, at least when using older radiation techniques (3, 6). In addition, the Nation Comprehensive Cancer Network (NCCN) clinical practice guidelines on NSCLC has recommended a minimum number of six nodes removed during surgical resection, three from N1 and three from N2 stations (3). Due to the uncertainty in surgical practice, the resected nodes may not achieve the required number. Since large tumor size is considered as a risk factor of mediastinal lymph nodes involvement even in early clinical stage lung cancer (7, 8), insufficient mediastinal lymph nodes evaluation may lead to a false-negative N descriptor. The consequent imprecise staging can probably misguide the therapeutic strategies, especially PORT, and lead to higher risk of recurrence and metastasis (9, 10). However, the value of PORT for node-negative large tumors has been frequently buried among plenty of studies on the impact of adjuvant therapy for the various stages of disease. With the rapid advance in radiation techniques in the past two decades, the role of PORT should be reevaluated.

The purpose of this study was to evaluate the effect of postoperative radiotherapy on long-term survival of patients with node-negative solitary large NSCLC within a large national database.

## Materials and Methods

### Patients Collection

This study was based on the SEER-18 registry databases, which currently covers ~28% of the United States population and routinely collects data on demographics, tumor sites, stage at diagnosis, first course of treatment, and follow-up of vital status. We identified the patients diagnosed with lung cancer based on the value of primary site variable (C34.0-34.9). Non-small cell lung cancer (NSCLC) patients was identified using the ICD-O-3 codes, histologic subgroups were defined as squamous cell carcinomas (8050-8052, 8070-8078), adenocarcinomas (8140-8147, 8250-8255, 8260, 8310, 8430, 8480, 8481, 8571-8575) and other types such as large cell carcinoma (8012-8013). The eligible criteria included: (1) diagnosed between 2004 and 2014 and lung was the first primary site, (2) age older than 18 years, (3) underwent surgery to the primary site and with a survival time ≥3 months, (4) CS tumor size 2004+ >5 cm and pathological stage T2b-3, N0, and M0 (according to the 7th edition of the AJCC staging manual), (5) cases with death certificate or autopsy were excluded. Types of primary surgery included sublobar resection, lobectomy, and pneumonectomy.

### Statistical Analysis

The variables in our analysis included age at diagnosis, gender, race, marital status, characteristics of tumor (location, size, histologic grade and type) and treatment to the primary site (surgical type, sequence of radiation, number of lymph nodes examined) and months of survival and vital status. Patients were divided into two groups: (1) surgery group; (2) surgery plus adjuvant radiotherapy (SART) group, depending on whether they received PORT or not. In order to minimize selection bias under the analytic settings with observational data, we performed a propensity score matching (PSM) analysis between patients with and without PORT based on age, race, and marital status, characteristics of tumor and surgery types. Due to the significantly different number of patients in two groups, a one-to-two matching was conducted based on the nearest neighbor method. Student's *t*-test was employed for continuous data, and we evaluated categorical variables using the Chi-square test of Fisher's exact test. A log-rank test was used to compare Kaplan-Meier survival curves. We defined the Overall survival (OS) as the time from the date of initial treatment to the date of death or the last day of follow-up. Cancer-specific survival (CSS) was measured from the data of initial treatment to death from NSCLC. For multivariate analyses in the matched population, we used the Cox proportional hazards model adjusting all the variables included in the study with *p*-value <0.2 in the univariate analyses. Two-sided *p*-value < 0.05 was considered as statistically significant. Hazard ratios with 95% confidence intervals were employed to quantify the strength of the association between predictors and survival. All analyses were performed with the IBM SPSS Statistics 22.0 (IBM, NY, United States), and images of statistics were produced using GraphPad Prism 7.0 (GraphPad Software, San Diego, CA, USA).

## Results

### General Information

Overall, the study cohort composed of 4,557 patients, of whom 526 patients (5.6%) underwent SART, as compared with 4,031 patients who underwent surgery alone (Table 1). The median follow-up time for the entire cohort was 29 (mean 39.6, range: 3–131). The mean age of the whole cohort was 67.1 years old (median, 68; range, 20–94 years old). Most patients were white in both groups (84.1 and 83.3%, respectively). Squamous cell carcinoma was the predominant histology type in the entire cohort, followed by adenocarcinoma. Notably, there were significant differences in patients' age, histology type, pathological grade, lobe distribution, and types of resection in both groups.

**Table 1:** Characteristics of patients in the entire cohort.

**Characteristics**	**Before PSM**			**After PSM**		
	**Surgery group**	**SART group**	***P***	**Surgery group**	**SART group**	***P***
	**(*n* = 4031)**	**(*n* = 526)**		**(*n* = 1042)**	**(*n* = 526)**	
**Gender (%)**			0.850			0.549
Men	2419 (60.0)	318 (60.5)		612 (58.7)	317 (60.4)	
Women	1612 (40.0)	208 (39.5)		430 (41.3)	208 (39.6)	
**Age, year**			<0.001			0.564
Mean ± SD	67.4 ± 10.1	64.90 ± 10.4		65.3 ± 10.6	64.9 ± 10.4	
Median(range)	68 (20–94)	66 (31-92)		66 (29-90)	66 (31-92)	
**Ethnicity (%)**			0.186			0.904
Caucasian	3390 (84.1)	438 (83.3)		858 (82.3)	437 (83.2)	
African	398 (9.9)	63 (12.0)		131 (12.6)	63 (12.0)	
Others	243 (6.0)	25 (4.8)		53 (5.1)	25 (4.8)	
**Marital status (%)**			0.539			
Married	2394 (59.4)	320 (60.8)		623 (59.8)	319 (60.8)	0.752
Unmarried	1637 (40.6)	206 (39.2)		419 (40.2)	206 (39.2)	
**Histology type (%)**			<0.001			
Squamous cell carcinoma	1633 (40.5)	252 (47.9)		499 (47.9)	251 (47.8)	0.994
Adenocarcinoma	1668 (41.4)	171 (32.5)		341 (32.7)	171 (32.6)	
Others	730 (18.1)	103 (19.6)		202 (19.4)	103 (19.6)	
**Pathological grade (%)**			<0.001			0.858
Well differentiated	456 (11.3)	28 (5.3)		63 (6.0)	28 (5.3)	
Moderately differentiated	1370 (34.0)	148 (28.1)		276 (26.5)	148 (28.2)	
Poorly differentiated/Undifferentiated	1962 (48.7)	313 (59.5)		631 (60.6)	312 (59.4)	
**Tumor size (cm)**			0.162			0.827
5-7 cm	2588 (64.2)	321 (61.0)		631 (60.6)	321 (61.1)	
>7 cm	1443 (35.8)	205 (39.0)		411 (39.4)	204 (38.9)	
**Location (%)**			0.765			0.549
Left	1620 (40.2)	207 (39.4)		426 (40.9)	206 (39.2)	
Right	2408 (59.7)	319 (60.6)		616 (59.1)	319 (60.8)	
**Lobe distribution (%)**			<0.001			0.895
Upper lobe	2080 (51.6)	361 (68.6)		709 (68)	361 (68.8)	
Middle Lobe	148 (3.7)	12 (2.3)		29 (2.8)	12 (2.3)	
Lower lobe	1603 (39.8)	121 (23.0)		236 (22.6)	121 (23)	
**Types of resection (%)**			<0.001			0.775
Sublobar resection	197 (4.9)	53 (10.1)		105 (10.1)	52 (9.9)	
Lobectomy	3464 (85.9)	442 (84.0)		866 (63.1)	442 (84.2)	
Pneumonectomy	370 (9.2)	31 (5.9)		71 (6.8)	31 (5.9)	
**Number of nodes examined**			<0.001			0.668
<6	1467 (36.4)	252 (47.9)		486 (46.6)	251 (47.8)	
≥6	2564 (63.6)	274 (52.1)		556 (53.4)	274 (52.2)	

*PSM, propensity-scored matching; SART, surgery plus adjuvant radiotherapy*.

To eliminate selection biases caused by such confounding factors, a 1:2 PSM was conducted between the SART group and surgery group. 1,042 and 525 cases in surgery group and SART group were finally matched for analysis (Table 1). There was no significant difference in any patient characteristics between two groups after matching. Multivariate regression analysis identified gender, age, histology type, differentiation grade, tumor size, SART, and number of examined lymph nodes as risk factors for OS. These risk factors were also found to significantly impact CSS except for histology type (Supplementary Table 1).

### Comparison of Treatment Modality

**Figure 1:**
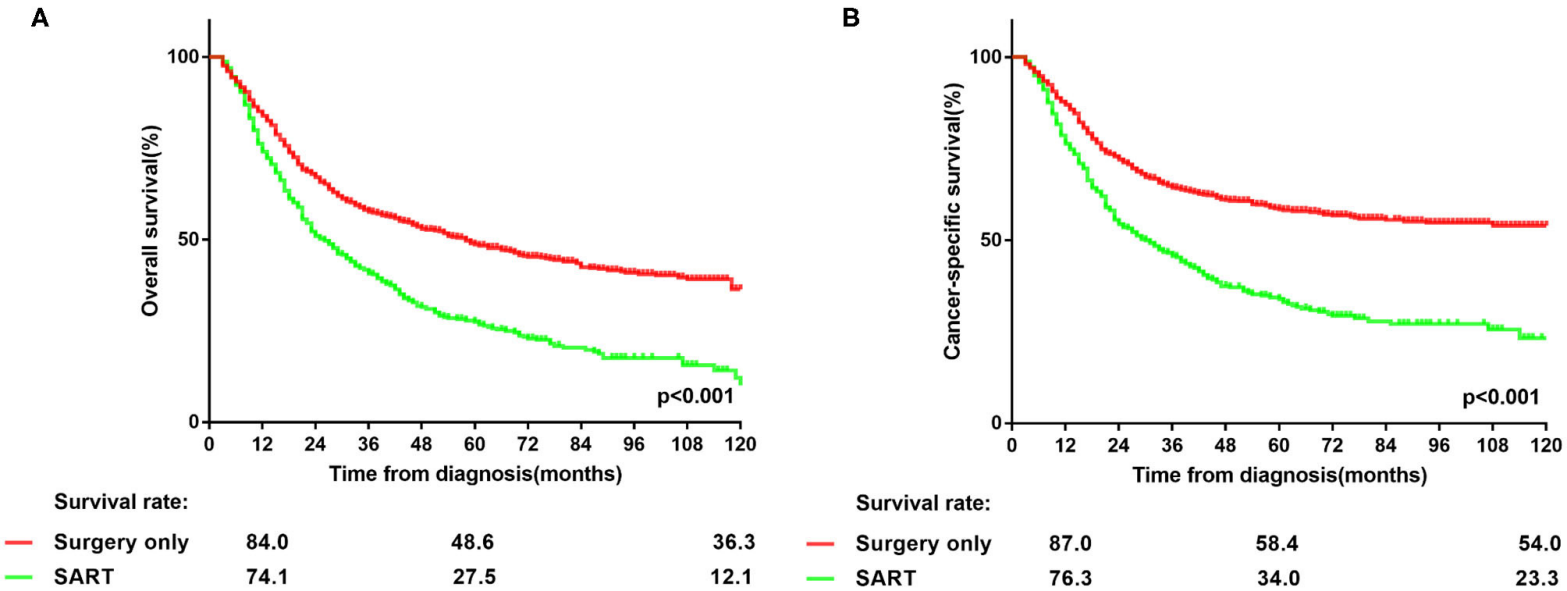
**(A,B)** Overall and lung cancer-specific survivals in patients with NSCLC >5 cm who underwent surgery alone or surgery plus adjuvant radiotherapy.

Notably, the majority of patients underwent surgery alone in the entire PSM cohort (Table 1). Lobectomy predominated in the types of resection in both groups (Table 1). As shown in Figure 1, patients in the surgery group had significantly better OS (*p* < 0.001) and CSS (*p* < 0.001) than those in SART group. In other word, surgery alone remained the primary option in the treatment of patients with NSCLC larger than 5 cm without lymph nodes involvement.

**Figure 2:**
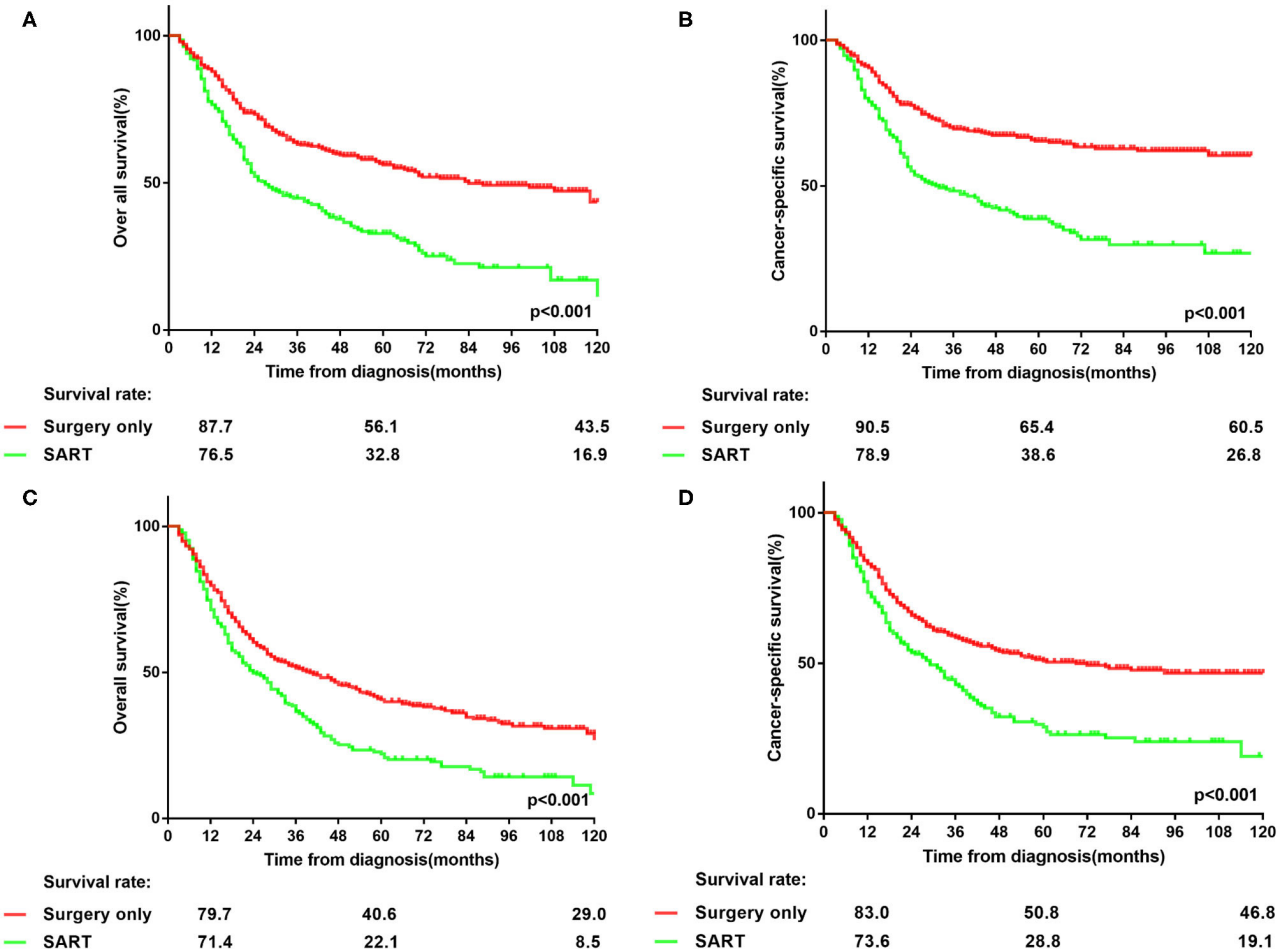
Stratification of overall survival and lung cancer-specific survival in patients with node-negative NSCLC >5 cm at the cut point of the number of harvested lymph nodes who underwent surgery or surgery plus adjuvant radiotherapy. **(A,B)** overall survival and lung cancer-specific survival in patients with node-negative NSCLC > 5 cm who had more than 6 lymph nodes dissected. **(C,D)** overall survival and lung cancer-specific survival in patients with node-negative NSCLC > 5 cm who had <6 lymph nodes examined.

Since insufficient examined lymph nodes can result in a false-negative N stage, the prognosis of patients in two groups was compared to investigate whether PORT can benefit patients with solitary large tumors, based on the stratification of the number of dissected lymph nodes. The cut-off value was set as six according to the NCCN guidelines (3). As shown in Figure 2, the prognosis of patients in surgery group was better than that in the other group (*p* < 0.001), irrespective of the number of examined lymph nodes. Moreover, in surgery group, patients with more lymph nodes examined showed better prognosis than those with nodes examined less than six (*p* < 0.001) (Supplementary Table 2). In contrast, more examined lymph nodes provided no remarkably additional survival benefit for patients in SART group but only a trend of prolonged OS (*p* = 0.052) and CSS (*p* = 0.115) (Supplementary Table 2). Therefore, PORT should not be recommended for node-negative NSCLC patients with tumor size > 5 cm.

Furthermore, a Cox proportional hazards regression model was applied to further study the potential risk factors in subgroups of NSCLC > 5 to 7 cm and > 7 cm (Table 2). In either subgroups, SART was associated with significantly decreased OS and CSS (OS with NSCLC > 5 to 7 cm: HR, 1.896; 95% CI: 1.573 to 2.285; *p* < 0.001; CSS with NSCLC > 5 to 7 cm: HR, 2.172; 95% CI: 1.755 to 2.689; *p* < 0.001; OS with NSCLC > 7 cm: HR, 1.635; 95% CI: 1.288 to 2.075; *p* < 0.001; CSS with NSCLC > 7 cm: HR, 1.751; 95% CI: 1.351 to 2.269; *p* < 0.001). Interestingly, number of lymph nodes dissected less than six was found to have a significantly adverse impact on OS (HR, 1.398; 95% CI: 1.162 to 1.68; *p* < 0.001) and CSS (HR, 1.462; 95% CI:1.18 to 1.811; *p* = 0.001) in patients with NSCLC > 5 to 7 cm, compared with more examined lymph nodes. Similar results for OS (HR, 0.748; 95% CI: 0.591 to 0.946; *p* = 0.015) and CSS (HR, 0.762; 95% CI: 0.589 to 0.986; *p* = 0.038) were observed in patients with NSCLC > 7 cm (Table 2).

**Table 2:** Cox proportional hazards regression model for overall survival and lung cancer–specific survival in patients with non–small-cell lung cancer > 5 to 7 cm and > 7 cm.

	**No. (%) of Patients by NSCLC Size and Survival Type in the matched group**							
	**> 5 to 7 cm**				**> 7 cm**			
	**Overall Survival**		**Cancer Specific Survival**		**Overall Survival**		**Cancer Specific Survival**	
**Variable**	**Hazard Ratio (95%CI)**	***P***	**Hazard Ratio (95%CI)**	***P***	**Hazard Ratio (95%CI)**	***P***	**Hazard Ratio (95%CI)**	***P***
**Gender**		0.269		0.903		0.003		0.009
Men	1.00 (reference)		1.00 (reference)		1.00 (reference)		1.00 (reference)	
Women	0.9 (0.746 to 1.085)		1.014 (0.817 to 1.257)		0.680 (0.527 to 0.879)		0.686 (0.518 to 0.909)	
**Age(y)**	1.025 (1.016 to 1.034)	0	1.016 (1.006 to 1.026)	0.002	1.029 (1.017 to 1.043)	0	1.022 (1.008 to 1.036)	0.002
**Ethnicity**	-		-		-		-	
Caucasian								
African								
Other								
**Marital status**	-		-			0.022		0.218
Married					1.00 (reference)		1.00 (reference)	
Unmarried					1.324 (1.042 to 1.683)		1.18 (0.906 to 1.537)	
**Histology type**		0.121		0.554		0.57		0.191
SCC	1.00 (reference)		1.00 (reference)		1.00 (reference)		1.00 (reference)	
ADC	0.83 (0.668 to 1.031)		0.928 (0.725 to 1.187)		0.939 (0.706 to 1.25)		1.009 (0.736 to 1.382)	
Others	1.073 (0.841 to 1.37)		1.1 (0.826 to 1.464)		1.132 (0.821 to 1.561)		1.344 (0.958 to 1.885)	
**Grade**		0.492		0.239		0.099		0.108
Well differentiated	1.00 (reference)		1.00 (reference)		1.00 (reference)		1.00 (reference)	
Moderately	1.314 (0.839 to 2.058)		1.584 (0.914 to 2.745)		1.57 (0.833 to 2.959)		1.556 (0.757 to 3.197)	
differentiated								
Poorly	1.264 (0.82 to 1.95)		1.57 (0.922 to 2.673)		1.853 (0.995 to 3.453)		1.905 (0.943 to 3.849)	
differentiated /								
Undifferentiated								
**Location**		0.006		0.005		0.199		0.073
Left	1.00 (reference)		1.00 (reference)		1.00 (reference)		1.00 (reference)	
Right	0.774 (0.644 to 0.931)		0.736 (0.595 to 0.91)		1.171 (0.92 to 1.49)		1.276 (0.978 to 1.665)	
**Lobe**	-		-			0.64		0.084
Upper					1.00 (reference)		1.00 (reference)	
Middle					1.292 (0.593 to 2.815)		1.821 (0.792 to 4.188)	
Lower					1.115 (0.843 to 1.475)		1.33 (0.985 to 1.795)	
**Sequence of radiation**		0		0		0		0
Surgery Alone	1.00 (reference)		1.00 (reference)		1.00 (reference)		1.00 (reference)	
SART	1.896 (1.573 to 2.285)		2.172 (1.755 to 2.689)		1.635 (1.288 to 2.075)		1.751 (1.351 to 2.269)	
**Number of LN**		0		0.001		0.015		0.038
<6	1.00 (reference)		1.00 (reference)		1.00 (reference)		1.00 (reference)	
≥6	0.699 (0.582 to 0.84)		0.674 (0.544 to 0.834)		0.748 (0.591 to 0.946)		0.762 (0.589 to 0.986)	

*SCC, squamous cell carcinoma; ADC, adenocarcinoma; SART, surgery plus adjuvant radiotherapy; LN, lymph nodes*.

Since the preferred role of surgery alone has been proved, the surgical procedures were compared to assess the optimal one in patients treated with surgery alone. Another Cox proportional hazards regression model was applied to confirm the impact on prognosis of different types of resection (Table 3). In patients with NSCLC > 5 to 7 cm, lobectomy and pneumonectomy, compared with sublobectomy, was associated with increased OS (OS with lobectomy vs. sublobectomy: HR, 0.596; 95% CI: 0.433 to 0.82; *p* = 0.002; OS with pneumonectomy vs. sublobectomy: HR, 1.023; 95% CI: 0.566 to 1.847; *p* = 0.093) and CSS (CSS with lobectomy vs. sublobectomy: HR, 0.525; 95% CI: 0.36 to 0.766; *p* = 0.001; CSS with pneumonectomy vs. sublobectomy: HR, 0.867; 95% CI: 0.427 to 1.759; *p* = 0.692). Meanwhile, lobectomy was associated with increased OS (HR, 0.583; 95% CI: 0.348 to 0.976, *p* = 0.040) and equal CSS (HR, 0.606; 95% CI: 0.325 to 1.131, *p* = 0.116) in patients with NSCLC > 5 to 7 cm. Therefore, lobectomy should be attempted as the optimal type of resection for patients with NSCLC > 5 to 7 cm. In terms of NSCLC > 7 cm, neither lobectomy nor pneumonectomy was associated with increased OS (OS with lobectomy vs. sublobectomy: HR, 0.842; 95% CI: 0.521 to 1.36; *p* = 0.482; OS with pneumonectomy vs. sublobectomy: HR, 0.921; 95% CI: 0.476 to 1.784; *p* = 0.807) and CSS (CSS with lobectomy vs. sublobectomy: HR, 0.922; 95% CI: 0.532 to 1.598; *p* = 0.773; CSS with pneumonectomy vs. sublobectomy: HR, 0.891; 95% CI: 0.411 to 1.931; *p* = 0.77) compared with sublobectomy. Thus sublobectomy might be considered as an alternative to lobectomy for patients with NSCLC > 7 cm who cannot tolerate lobectomy. In addition, for patients who underwent only surgery, multivariate regression analysis identified age and number of examined lymph nodes as significant prognostic factors (Table 3).

**Table 3:** Cox proportional hazards regression model for overall survival and lung cancer–specific survival in patients with non–small-cell lung cancer > 5 to 7 cm and > 7 cm who underwent surgery alone.

	**No. (%) of Patients by NSCLC Size and Survival Type in the matched group**							
	**> 5 to 7 cm**				**> 7 cm**			
	**Overall Survival**		**Cancer-Specific Survival**		**Overall Survival**		**Cancer-Specific Survival**	
**Variable**	**Hazard Ratio (95%CI)**	***P***	**Hazard Ratio (95%CI)**	***P***	**Hazard Ratio (95%CI)**	***P***	**Hazard Ratio (95%CI)**	***P***
**Gender**		0.022		0.257		0.014		0.066
Men	1.00 (reference)		1.00 (reference)		1.00 (reference)		1.00 (reference)	
Women	0.756 (0.596 to 0.961)		0.847 (0.636 to 1.129)		0.675 (0.493 to 0.925)		0.722 (0.51 to 1.022)	
**Age(y)**	1.031 (1.02 to 1.044)	0	1.018 (1.004 to 1.032)	0.012	1.028 (1.014 to 1.043)	0	1.018 (1.002 to 1.033)	0.026
**Ethnicity**	-		-		-		-	
Caucasian								
African								
Other								
**Marital status**	-		-			0.026		0.183
Married					1.00 (reference)		1.00 (reference)	
Unmarried					1.399 (1.041 to 1.879)		1.251 (0.9 to 1.738)	
**Histology type**	-		-			0.536		0.395
SCC					1.00 (reference)		1.00 (reference)	
ADC					0.847 (0.594 to 1.206)		0.859 (0.577 to 1.279)	
Others					1.047 (0.718 to 1.525)		1.168 (0.778 to 1.754)	
**Grade**	-		-		-		-	
Well differentiated								
Moderately								
differentiated								
Poorly								
differentiated /								
Undifferentiated								
**Location**	-		-			0.77		0.593
Left					1.00 (reference)		1.00 (reference)	
Right					1.046 (0.773 to 1.417)		1.096 (0.783 to 1.535)	
**Lobe**	-		-			0.519		0.422
Upper					1.00 (reference)		1.00 (reference)	
Middle					1.632 (0.696 to 3.827)		1.856 (0.73 to 4.717)	
Lower					0.988 (0.705 to 1.384)		1.068 (0.741 to 1.539)	
**Number of LN examined**		0.003		0.009		0.026		0.02
<6	1.00 (reference)		1.00 (reference)		1.00 (reference)		1.00 (reference)	
≥6	0.698 (0.550 to 0.886)		0.677 (0.506 to 0.906)		0.716 (0.533 to 0.961)		0.678 (0.489 to 0.941)	
**Surgery type**		0.001		0.001		0.755		0.949
Wedge resection	1.00 (reference)		1.00 (reference)		1.00 (reference)		1.00 (reference)	
Lobectomy	0.596 (0.433 to 0.82)		0.525 (0.36 to 0.766)		0.842 (0.521 to 1.36)		0.922 (0.532 to 1.598)	
Pneumonectomy	1.023 (0.566 to 1.847)		0.867 (0.427 to 1.759)		0.921 (0.476 to 1.784)		0.891 (0.411 to 1.931)	

*SCC, squamous cell carcinoma; ADC, adenocarcinoma; LN, lymph nodes*.

## Discussion

Despite the increasing detection rate of early-stage NSCLC present as small pulmonary nodules, locally advanced NSCLC remain a complicated and thorny problem in clinical practice. For very large tumors, most clinicians would consider that the optimal treatment modality is still undefined. Part of the confusion arises from the reclassification of T2b tumors > 5 cm to T3 tumors and subsequent changes to stage groupings involving T3 tumors > 5 cm from stage IIA to IIB if node-negative (4, 11). Complete resection is still considered the optimal treatment for locally advanced disease with or without adjuvant chemotherapy to reduce the risk of distant recurrence (3, 12). Furthermore, treatment of stage IIIA disease including T4N0 may include determination of resectability as part of a multidisciplinary consultation (3).

Radiotherapy has been defined a role before or after surgery for locally advanced NSCLC (3), especially for microscopic residual disease (13). However, the latest NCCN guidelines has also pointed out that PORT is not recommended for patients with pathologic stage N0-1 disease at least when using older radiation techniques (3, 6), because it has been associated with increased mortality. Although the cited clinical evidence ranked the highest level, the source itself was a meta-analysis published in 2005. However, the radiotherapy planning underwent major changes during the past decades (14). The radiation techniques has also stridden forward from the era of two- dimension (2D) to three-dimension (3D) with 3D-conformal radiotherapy (3D-CRT), intensity modulated radiotherapy (IMRT) (14, 15), stereotactic body radiation therapy (SBRT) and the latest proton radiotherapy widely applied within 2004-2014. Therefore, whether the updated radiation techniques can additionally benefit postoperative patients awaits a definite answer. Hitherto, there have been neither radiotherapists nor surgeons focusing on the role of PORT with the new generation of radiation techniques for N0 advanced-stage disease. In our study, it has been well demonstrated that the survival advantages favor surgery alone rather than SART for NSCLC > 5 cm to 7 cm and > 7 cm. The results further validated the prior role of surgery for treating large pulmonary malignancy without nodal involvement.

A larger tumor size indicated potentially higher risk of occult lymph nodes metastasis (16–18) and micrometastasis (19, 20) in clinical N0 disease. Recent researches also indicated the residual malignant cells in lymph nodes plays a role in recurrence and distant metastasis (21, 22). Therefore, it is a reasonable assumption that PORT might benefit postoperative patients to some extent. However, evidence from our study conflicts with that logic and suggests the undoubted position of complete surgical resection. Another retrospective study published in 2006 using the SEER database drew a similar conclusion that PORT is associated with a decrease in survival in patients with N1 and N0 nodal disease. Additionally, due to the recommendation proposed by the AJCC, UICC, and IASLC that at least six nodes should be removed during surgical resection (three from N1 and three from N2 stations), great interest has been raised about whether there could be any difference in the prognosis of patients with NSCLC > 5 to 7 cm and NSCLC > 7 cm based on the suggested number of examined lymph nodes. Our data revealed that the superiority of examining more than six lymph nodes extends to both subgroups. Although more examined lymph nodes led to non-significant improvement on the prognosis of patients who underwent SART, it could be possibly attributed to the local control on residual lymph nodes by PORT. Actually, the long-term survival benefit of more examined lymph nodes on patients has already been reported by Liang et al. (10), who recommended 16 lymph nodes as the cut point for evaluating the quality of lymph nodes examination or prognostic stratification postoperatively for patients with declared node-negative disease. Therefore, our data kept consistent with their findings and supported the value of a thorough lymph nodes examination in NSCLC > 5 cm.

Tumor size has been recognized as a significant prognostic factor of survival outcomes, particularly in patients with early-stage NSCLC (4, 12). Morgensztern et al. (23) previously demonstrated that tumor size is an independent predictor of overall and lung cancer-specific survival in patients with locally advanced disease as well. In our study, tumor size was also associated with a higher risk of decreased OS and CSS upon multivariate analysis. Nowadays, lobectomy has been recommended as the standard surgical procedure for operable NSCLC (3, 24), especially for tumors larger than 2 cm (25–27). Based on our data, lobectomy should be considered as the first choice for NSCLC > 5 to 7 cm which was congruent with the current guidelines. However, lobectomy may be not suitable for NSCLC > 7 cm, at least not superior over sublobectomy in our study. It seemed that for patients who could not tolerate lobectomy with NSCLC > 7 cm, sublobectomy should be recommended as an optimal alternative surgical procedure. In fact, large NSCLC sometimes invade neighboring structures and possibly result in R1 or R2 resections even with lobectomy. Therefore, increasing tumor size could partly account for the non-significant difference in OS and CSS between patients who underwent lobectomy and sublobectomy in patients with NSCLC > 7 cm. A study by Dziedzic et al. (9) identified risk factors for recurrence including tumor size of 5-7 cm and > 7 cm, which partially supported our results. However, both sublobar resection and pneumonectomy were proved to associate with local and distant recurrence (9) which conflicted our data. The disparity may be attributed to the evaluation of appropriate surgical procedures based on stratification of tumor size in our study. To be cautious, we believe that high-quality evidence from ongoing randomized controlled trials are needed to verify our results.

We must acknowledge some limitations of this study. First, potential biases were inevitable because of the retrospective nature of this study. Though some advanced statistical methods were applied to balance the covariates among the arms, there were still some latent biases that could not be adjusted. For example, there was no information on anatomical location and pulmonary function which can affect the types of resection. Furthermore, the information absence of resection margin also poses an insurmountable obstacle for our study, since R1 and R2 resection often led to subsequent PORT and probably resulted in a worse prognosis. Meanwhile, there were potential biases on the prognostic impact of the number of examined lymph nodes because of the lack of definite lymph nodes stations and whether en-bloc resection was performed. In the SEER database there is no ability to discern which patients with tumors > 5 cm received adjuvant chemotherapy, therefore either group invariably included this subset of patients. Notably, information regarding the administration of chemotherapy, either as neoadjuvant or adjuvant therapy, is unavailable in the SEER database as well. Therefore, we could not comprehensively analyze the influence of neoadjuvant chemotherapy alone or adjuvant chemotherapy when used concurrently with radiotherapy on long-term survival of patients with NSCLC > 5 cm. Additionally, no information regarding radiation techniques, including total dose, fraction size, and beam energy, was available, and therefore was not accounted in our analysis. Variations in adjuvant chemotherapy and radiotherapy regimens are likely to be confounded in our study population and may have influenced the lack of significant PORT benefit on survival over pulmonary resection alone.

In conclusion, surgery alone should be recommended as the first choice for patients with NSCLC > 5 cm. The number of examined lymph nodes should be more than six in patients with NSCLC > 5 cm, especially for those who undergo surgery alone. For patients with NSCLC > 7 cm who could not tolerate lobectomy, sublobectomy might be an optimal alternative surgical procedure.

## Data Availability Statement

Publicly available datasets were analyzed in this study. This data can be found here: https://seer.cancer.gov/seerstat/.

## Ethics Statement

The SEER database is available to the public and all patient identities are protected. Our study was therefore exempted from institutional review board at our hospital.

## Author Contributions

BW, YZ, MJ, and JW are lead authors who participated in data collection, manuscript drafting, table/figure creation, and manuscript revision. BW and YZ aided in data collection. JW and RW is the corresponding author who initially developed the concept and drafted and revised the manuscript. All authors read and approved the final manuscript.

## Conflict of Interest

The authors declare that the research was conducted in the absence of any commercial or financial relationships that could be construed as a potential conflict of interest.

## Abbreviations

NSCLC: non-small cell lung cancer
OS: overall survival
CSS: cancer–specific survival
IASLC: the International Association for the Study of Lung Cancer
AJCC: the American Joint Committee on Cancer
UICC: the Union for International Cancer Control
PORT: postoperative radiotherapy
NCCN: the Nation Comprehensive Cancer Network
SART: surgery plus adjuvant radiotherapy
PSM: propensity score matching.
