# Supplementary material for: Choice of Treatment for Patients With Non–small-cell Lung Cancer >5 cm Between Surgery Alone and Surgery Plus Adjuvant Radiotherapy
Source: Front Surg. 2021 Mar 9;8:649802. doi: 10.3389/fsurg.2021.649802 (PMC7985264; doi:10.3389/fsurg.2021.649802)
Supplement: Supplementary file 1 [file Data_Sheet_1.docx]

| **Supplementary Table 1** **Univariate and Multivariate Analysis on Risk Factors of Overall and Lung Cancer-Specific Survival for Overall Cohort.** | | | | | | | | |
| --- | --- | --- | --- | --- | --- | --- | --- | --- |
| **Characteristics** | **Overall Survival** | | | | **Cancer-Specific Survival** | | | |
|  |  |  |  |  |  |  |  |  |
|  | **Univariate analysis** | | **Multivariate analysis** | | **Univariate analysis** | | **Multivariate analysis** | |
|  | **Hazard Ratio**  **(95%CI)** | ***P*** | **Hazard Ratio**  **(95%CI)** | ***P*** | **Hazard Ratio**  **(95%CI)** | ***P*** | **Hazard Ratio**  **(95%CI)** | ***P*** |
| **Sex** |  | 0 |  | 0.004 |  | 0.02 |  | 0.044 |
| Men | 1.00(reference) |  | 1.00(reference) |  | 1.00(reference) |  | 1.00(reference) |  |
| Women | 0.779(0.678 to 0.895) |  | 0.808(0.698 to 0.935) |  | 0.824(0.7 to 0.97) |  | 0.844(0.716 to 0.995) |  |
| **Age(y)** | 1.025(1.018 to 1.031) | 0 | 1.027(1.02 to 1.035) | 0 | 1.017(1.009 to 1.025) | 0 | 1.02(1.012 to 1.028) | 0 |
| **Ethnicity** |  | 0.127 |  | 0.09 |  | 0.175 |  | 0.207 |
| Caucasian | 1.00(reference) |  | 1.00(reference) |  | 1.00(reference) |  | 1.00(reference) |  |
| African | 0.911(0.739 to 1.122) | 0.38 | 0.951(0.759 to 1.191) | 0.66 | 0.914(0.716 to 1.166) | 0.469 | 0.979(0.763 to 1.257) | 0.869 |
| Other | 0.71(0.499 to 1.012) | 0.058 | 0.665(0.461 to 0.96) | 0.029 | 0.686(0.452 to 1.042) | 0.077 | 0.69(0.458 to 1.04) | 0.076 |
| **Marital status** |  | 0.23 |  | |  | 0.702 |  | |
| Married | 1.00(reference) |  |  |  | 1.00(reference) |  |  |  |
| Unmarried | 1.087(0.948 to 1.247) |  |  |  | 1.032(0.877 to 1.214) |  |  |  |
| **Histology type** |  | 0.014 |  | 0.016 |  | 0.106 |  | 0.051 |
| Squamous Cell Carcinomas | 1.00(reference) |  | 1.00(reference) |  | 1.00(reference) |  | 1.00(reference) |  |
| Adenocarcinomas | 0.807(0.69 to 0.943) | 0.007 | 0.899(0.761 to 1.062) | 0.21 | 0.863(0.719 to 1.037) | 0.116 | 0.972(0.806 to 1.172) | 0.767 |
| Others | 1.015(0.853 to 1.207) | 0.869 | 1.214(1.003 to 1.468) | 0.046 | 1.085(0.884 to 1.331) | 0.437 | 1.265(1.021 to 1.567) | 0.031 |
| **Grade** |  | 0.087 |  | 0.011 |  | 0.037 |  | 0.006 |
| Well differentiated | 1.00(reference) |  | 1.00(reference) |  | 1.00(reference) |  | 1.00(reference) |  |
| Moderately differentiated | 1.444(1.021 to 2.044) | 0.038 | 1.597(1.119 to 2.28) | 0.01 | 1.603(1.062 to 2.42) | 0.025 | 1.77(1.162 to 2.697) | 0.008 |
| Poorly differentiated/Undifferentiated | 1.453(1.041 to 2.029) | 0.028 | 1.7(1.203 to 2.403) | 0.003 | 1.68(1.13 to 2.498) | 0.01 | 1.936(1.285 to 2.916) | 0.002 |
| **Tumor size** |  | 0.025 |  |  |  | 0.001 |  | 0 |
| 5-7cm | 1.00(reference) |  | 1.00(reference) |  | 1.00(reference) |  | 1.00(reference) |  |
| >7cm | 1.168(1.019 to 1.339) |  | 1.247(1.079 to 1.44) | 0.003 | 1.302(1.109 to 1.527) |  | 1.38(1.174 to 1.623) |  |
| **Location** |  | 0.559 |  | |  | 0.736 |  | |
| Left | 1.00(reference) |  |  |  | 1.00(reference) |  |  |  |
| Right | 0.96(0.838 to 1.1) |  |  |  | 0.974(0.835 to 1.136) |  |  |  |
| **Lobe** |  | 0.582 |  |  |  | 0.556 |  |  |
| Upper | 1.00(reference) |  |  |  | 1.00(reference) |  |  |  |
| Middle | 1.151(0.752 to 1.763) | 0.517 |  |  | 1.204(0.751 to 1.929) | 0.44 |  |  |
| Lower | 1.074(0.914 to 1.262) | 0.383 |  |  | 1.081(0.901 to 1.297) | 0.403 |  |  |
| **Surgery type** |  | 0 |  | 0.019 |  | 0.008 |  | 0.063 |
| Wedge resection | 1.00(reference) |  | 1.00(reference) |  | 1.00(reference) |  | 1.00(reference) |  |
| Lobectomy | 0.642(0.525 to 0.785) | 0 | 0.757(0.605 to 0.947) | 0.015 | 0.697(0.547 to 0.888) | 0.004 | 0.752(0.584 to 0.968) | 0.027 |
| Pneumonectomy | 0.767(0.563 to 1.045) | 0.093 | 0.953(0.675 to 1.347) | 0.787 | 0.879(0.593 to 1.303) | 0.52 | 0.884(0.597 to 1.308) | 0.536 |
| **Sequence of radiation** |  | 0 |  | 0 |  | 0 |  | 0 |
| Surgery Alone | 1.00(reference) |  | 1.00(reference) |  | 1.00(reference) |  | 1.00(reference) |  |
| SART | 1.753(1.53 to 2.01) |  | 1.769(1.533 to 2.041) |  | 1.983(1.691 to 2.325) |  | 1.913(1.63 to 2.245) |  |
| **Number of nodal examined** |  | 0 |  | 0 |  | 0 |  | 0 |
| <6 | 1.00(reference) |  | 1.00(reference) |  | 1.00(reference) |  | 1.00(reference) |  |
| >=6 | 0.711(0.622 to 0.814) |  | 0.737(0.638 to 0.851) |  | 0.734(0.626 to 0.86) |  | 0.737(0.626 to 0.867) |  |
| Abbreviations: SART, surgery plus adjuvant radiotherapy. | | | | | | | | |

| **Supplementary Table 2 The Overall Survival and Lung Cancer-Specific Survival after Surgery Stratified by Number of Examined Lymph Nodes** | | | | | | | | | | | | | |
| --- | --- | --- | --- | --- | --- | --- | --- | --- | --- | --- | --- | --- | --- |
|  | No. (%) of Patients by Years after Surgery and Survival Type | | | | | | | | | | | | |
|  | Overall Survival | | | | | Cancer-Specific Survival | | | | | | | |
| Subgroups | 1(y) | 3(y) | 5(y) | 10(y) | ***P*** | 1(y) | | 3(y) | | 5(y) | | 10(y) | ***P*** |
| Surgery Alone |  |  |  |  | <0.001 |  |  | |  | |  | | <0.001 |
| < 6 | 79.7 | 51.5 | 40.6 | 29 |  | 83 | 58.5 | | 50.8 | | 46.8 | |  |
| ≥ 6 | 87.7 | 63.1 | 56.1 | 43.5 |  | 90.5 | 69.5 | | 65.4 | | 60.5 | |  |
| SART |  |  |  |  | 0.052 | 1 | 3 | | 5 | | 10 | | 0.115 |
| < 6 | 71.4 | 36.5 | 22.1 | 8.5 |  | 73.6 | 42.6 | | 28.8 | | 19.1 | |  |
| ≥ 6 | 76.5 | 44.7 | 32.8 | 16.9 |  | 78.9 | 48.3 | | 38.6 | | 26.8 | |  |
| Abbreviations: SART, surgery plus adjuvant radiotherapy. | | | | | | | | | | | | | |
